# Reaching Adolescent Gay, Bisexual, and Queer Men Online: Development and Refinement of a National Recruitment Strategy

**DOI:** 10.2196/jmir.5602

**Published:** 2016-08-04

**Authors:** Tonya L Prescott, Gregory Phillips II, L. Zachary DuBois, Sheana S Bull, Brian Mustanski, Michele L Ybarra

**Affiliations:** ^1^ Center for Innovative Public Health Research San Clemente, CA United States; ^2^ Department of Medical Social Sciences Feinberg School of Medicine Northwestern University Chicago, IL United States; ^3^ Department of Anthropology California State University Long Beach Long Beach, CA United States; ^4^ Community and Behavioral Health Colorado School of Public Health University of Colorado Denver, CO United States

**Keywords:** Facebook, mHealth, recruitment methods, intervention development, HIV, adolescent, AGBM, sexual minority

## Abstract

**Background:**

Using social networking websites to recruit research participants is increasingly documented in the literature, although few studies have leveraged these sites to reach those younger than 18 years.

**Objective:**

To discuss the development and refinement of a recruitment protocol to reach and engage adolescent gay, bisexual, and other teenaged men who have sex with men (AGBM). Participants were recruited for development and evaluation activities related to Guy2Guy, a text messaging–based human immunodeficiency virus infection prevention program.

**Methods:**

Eligibility criteria included being between 14 to 18 years old; being a cisgender male; self-identifying as gay, bisexual, and/or queer; being literate in English, exclusively owning a cell phone, enrolled in an unlimited text messaging plan, intending to keep their current phone number over the next 6 months, and having used text messaging for at least the past 6 months. Recruitment experiences and subsequent steps to refine the Internet-based recruitment strategy are discussed for 4 research activities: online focus groups, content advisory team, beta test, and randomized controlled trial (RCT). Recruitment relied primarily on Facebook advertising. To a lesser extent, Google AdWords and promotion through partner organizations working with AGBM youth were also utilized.

**Results:**

Facebook advertising strategies were regularly adjusted based on preidentified recruitment targets for race, ethnicity, urban-rural residence, and sexual experience. The result was a diverse sample of participants, of whom 30% belonged to a racial minority and 20% were Hispanic. Facebook advertising was the most cost-effective method, and it was also able to reach diverse recruitment goals: recruitment for the first focus group cost an average of US $2.50 per enrolled participant, and it took 9 days to enroll 40 participants; the second focus group cost an average of US $6.96 per enrolled participant, and it took 11 days to enroll 40 participants. Recruitment for the first content advisory team cost an average of US $32.52 per enrolled participant; the second cost US $29.52 per participant. Both recruitment drives required 10 days to enroll 24 participants. For the beta test, recruitment cost an average of US $17.19 per enrolled participant, and it took 16 days to complete enrollment of 20 participants. For the RCT, recruitment cost an average of US $12.54 per enrolled participant, and it took 148 days to enroll 302 participants. Google AdWords campaigns did not result in any enrolled participants of whom the research staff members were aware.

**Conclusions:**

Internet-based strategies can be a cost-efficient means to recruit and retain hard-to-reach populations from across the country. With real-time monitoring of participant demographic characteristics, diverse samples can be achieved. Although Facebook advertising was particularly successful in this study, alternative social media strategies can be explored in future research as these media are ever-changing.

## Introduction

Among the youth in the United States, 9 in 10 new human immunodeficiency virus (HIV) infections occur among adolescent gay, bisexual, and other teen men who have sex with men (AGBM) between the ages of 13-19 years [1]. Clearly, to affect HIV incidence, efforts to reach and engage AGBM are critical. In the face of this obvious need, it is perhaps surprising that few validated prevention programs are available [2]. In part, this lack of health programming reflects the challenge of successfully recruiting and retaining these hard-to-reach youth. More specifically, these challenges to recruitment and retention include the need to obtain parental permission or an institutional review board (IRB) waiver for those younger than 18 years [3]; gaining the trust of youth who may face social stigma for their sexual minority status; and recruiting a sufficient sample size, particularly if the sampling frame is local rather than national. Internet-based recruitment has the potential to address some of these challenges, but it can also introduce challenges in terms of validating the respondent and ensuring a diverse sample. Certainly with the popularity of social networking sites, particularly Facebook (FB), among adolescents [4], researchers are beginning to use Web-based recruitment strategies to engage hard-to-reach populations [5-11]. Even with the increasing use of other social networking sites (eg, Twitter, Instagram, Vine) FB remains the most popular site with youth, including 72% of adolescent men [4]. Furthermore, among youth who use social networking sites, 1 in 3 report using FB exclusively. Importantly, too, racial and ethnic minority youth are well represented among FB users. Usage is relatively similar by urbanicity (ie, 77% of urban, 75% of rural, 67% of suburban teenagers) and socioeconomic status (eg, 77% of youth from households earning less than US $50,000 annually compared with 68% of those from households earning more than US $50,000 annually) as well [4]. Given this diversity and breadth of reach, particularly among hard-to-reach youth, social networking sites may be an ideal place for researchers to recruit study participants.

Although the use of social networking websites to recruit research participants is increasingly documented in the literature [12], few studies have leveraged social networking websites to specifically reach *children* (ie, 18 years of age and younger). We also were unable to find any studies that documented the recruitment experiences of sexual minority youth in particular. To address this research gap, we will discuss our experiences developing and refining a national Internet-based recruitment protocol targeting AGBM 18 years of age and younger, primarily through FB advertising and also with Google AdWords and assistance from lesbian, gay, bisexual, and transgender (LGBT)–focused organizations. Participants were recruited for development and evaluation activities related to Guy2Guy, a text messaging–based HIV prevention and healthy sexuality program for AGBM. Lessons learned here have the potential to inform future HIV prevention programs aiming to recruit hard-to-reach populations, particularly sexual minority children.

## Methods

The research protocol was reviewed and approved by Chesapeake Institutional Review Board, the Center for Innovative Public Health Research’s IRB of record, and the Northwestern University IRB. Youth provided informed assent or consent, depending on their age, and completed a capacity to consent assessment [13-15]. A waiver of parental permission for participants younger than 18 years was obtained, primarily because requiring parental consent could increase risk to participants who may be victimized by their parents as a result of disclosing their sexual minority status [16]. The waiver also avoided the potential for fatal sampling bias that might occur if only youth who were out to their parents chose to enroll [17]. In addition to a waiver of parental permission, a Certificate of Confidentiality [18] was obtained from the National Institutes of Health to protect participants’ data from subpoenas and other law enforcement efforts.

Given the wide reach of both social media and text messaging, we developed a recruitment plan that facilitated enrollment of youth across the United States. The recruitment protocol was refined across 4 different national research activities that were delivered either online or via text messaging. These research activities included online focus groups to better understand AGBM sexual decision making, online content advisory teams to vet the proposed program messages, a beta test of the Guy2Guy text messaging program, and the randomized controlled trial (RCT) of Guy2Guy. Here, we describe the recruitment experiences and resulting recruitments of our strategy across time and research activities.

### Eligibility Criteria

Eligibility criteria for all research activities included being 14-18 years of age; being cisgender men (ie, those whose current gender identity and sex assigned at birth are both male); self-identifying as gay, bisexual, and/or queer; and being English-speaking. To promote the likelihood of the sample reflecting those who might use the text messaging–based program if it were publicly available, participants were also required to be exclusive owners of a cell phone, be enrolled in an unlimited text messaging plan, intend to keep their current phone number for the next 6 months, and have used text messaging for at least the past 6 months. Exclusion criteria included knowing another person already enrolled in the program and participating in a previous study activity (eg, those who took part in the focus groups were ineligible to take part in the RCT). The same eligibility criteria were applied for each study development activity with one exception: male gender identity was directly queried after the focus groups.

### Recruitment

Participants were recruited primarily using FB ads, which must adhere to character limits (ie, 25 characters for the headline, 90 characters for the body text) and include an image. These ads must also be approved by FB. Our ads were targeted based on the user’s location (United States), age (14-18 years), and sex (note “gender” is the term used by FB; male). We also included a list of 65 keywords (referred to as “interests” in the FB ad manager) relevant to AGBM including pop culture interests (eg, LGBT community, Katy Perry). Two types of ads were displayed depending on how the user accessed FB: News Feed ads (ie, ads are embedded in the dynamic news field central column) and right column ads (ie, ads are displayed in the static column on the right side of the webpage). People accessing FB on their desktop computer received both types of ads; those accessing the social networking site on a mobile device were only shown News Feed ads.

During the online focus groups, we asked partner organizations working with AGBM youth to promote the study. In subsequent research activities, we also used Google AdWords, which grants free advertising to the Center for Innovative Public Health Research for being a nonprofit organization. These ads included a title line of up to 25 characters, 2 description lines with up to 35 characters each, and a URL field. Ads linked to the project website, which included a brief project description and a Web-based contact form for those who wanted to be contacted for potential participation. The contact form was purposefully brief to reduce burden and promote form completion. As such, it consisted of 15 questions that queried study eligibility questions as well as demographic characteristics that were used to facilitate the recruitment of a diverse sample.

### Enrollment

Web-based eligibility screeners received were emailed to the project coordinator. Ineligible candidates were sent a link to a sexual health website [19] relevant to sexual minority youth via email. Eligible candidates and those whose eligibility was unclear (ie, because of “do not want to answer” responses) were sent a text message by research staff to schedule an appointment via telephone. On the call, research staff confirmed the candidate’s eligibility, explained the study, conducted a decisional capacity assessment [13-15], and obtained verbal assent or consent to participate. Those in the beta test and RCT also completed a self-safety assessment to determine whether it was safe for them to receive text messages about sensitive topics (eg, anal sex, being gay or bisexual) on their cell phones. The assent form, which included contact information for the IRB and principal investigator, was emailed to participants for later reference. All study recruitment and enrollment documents (eg, Web-based screener, assent or consent forms, self-safety assessment) can be found online [20]. For the focus groups and content advisory teams, enrollment occurred at the time of verbal assent or consent. Participants in the beta test and RCT were enrolled after they completed the baseline survey and confirmed receipt of a text message from the study program to ensure that their phone was compatible with the study software.

To ensure a diverse sample for the beta test and RCT, recruitment goals were preidentified by creating allocation “bins” (ie, recruitment goals) for race, ethnicity, urban-rural residence, and sexual experience (ie, ever versus never had anal or vaginal sex). Race and ethnicity targets were based on the 2010 US population demographics, which were the most current data available at the time of recruitment (ie, 72.4% white, 12.6% black, 16.3% Hispanic) [21]. Our targets were revised slightly so that the sample had a greater percentage of minority race (35%) and Hispanic ethnicity (20%). We also targeted 50% of the sample to be sexually inexperienced (ie, never had anal or vaginal sex) and 20% living in a rural residence. Urban-rural residence targets were identified to ensure sufficient representation among youth living in outer communities that often have fewer available LGBT resources. A Web-based study interface was programmed to automatically track the demographic characteristics of enrolled participants, allowing staff to monitor in real time the characteristics of the sample. Youth were enrolled sequentially until their bin was filled.

### Protecting Against Deception by Participants When Recruiting Online

Several measures were taken to limit the potential for deception by participants during the recruitment and enrollment process (ie, the same participant enrolling in the study multiple times, an ineligible candidate providing fake responses to become eligible), as this is often a concern when conducting Web-based research in which researchers are unable to see the candidate face-to-face [22]. The project description provided in the Web-based screener did not include study eligibility criteria or mention an incentive. Furthermore, to reduce a candidate’s ability to identify and provide the “right” answers for eligibility, the eligibility form did not exclusively include questions necessary to determine study eligibility, but also included questions to target enrollment (eg, race, ZIP code, sexual experience) and additional questions (eg, how they found the website). The eligibility screener also captured the candidate’s Internet protocol address, which allowed us to identify possible duplicate entries. Additionally, eligibility questions were queried again over the phone and compared with the answers provided previously on the Web-based screener. Discrepant responses for questions expected to be consistent (eg, age) were questioned further, whereas discrepant responses for more fluid questions (eg, sexual identity) were not further explored.

### Measures

The outcome measures for this enrollment case study included the following.

#### Advertisement Metrics

Quantification of study interest was based on measures provided by the FB and Google AdWords analytics reporting. “Clicks” referred to the number of total clicks on the ad. “Reach” referred to the number of people the ad was shown to. “Unique clicks” referred to the number of unique people who clicked on the ad. “Click-through rate” (CTR) referred to the number of clicks received divided by the number of impressions (ie, number of times the ad was shown). “Unique click-through rate” (uCTR) referred to the number of unique clicks received divided by the number of unique people the ad reached. The “average cost per click” (CPC) was calculated as the amount spent advertising divided by the CTR.

#### Recruitment Efficiency

The length of time to recruit the target sample and the number of youth who completed a screener needed to successfully enroll an eligible participant were indicators of recruitment efficiency. For the RCT, we also reported the number of phone calls required to enroll 1 person to quantify recruitment efficiency.

#### Sample Diversity

Because health disparities are apparent by race and ethnicity [1] and by rural versus urban community residence (as youth in rural communities often lack access to LGBT resources compared with those in urban settings), we believed it critically important to ensure the enrollment of a diverse sample. As such, we had a complex sampling scheme based on race, ethnicity, urban versus rural residence (determined by ZIP code), age, and sexual identity. For the sake of parsimony, we report the sample characteristics for the RCT only.

## Results

### Online Focus Groups

A total of 4 focus groups were conducted to inform the development of program content and the protocol: 2 with sexually experienced youth and 2 with sexually inexperienced youth. A more detailed description of the focus group methods and results are described elsewhere [23-25].

#### Advertisement Metrics

Facebook ads for the first round of focus groups (ie, 1 with sexually experienced youth, 1 with sexually inexperienced youth) were submitted to FB for approval on November 9, 2012 and approved the same day. The ads ran for 4 days (ie, November 9-12). On the basis of FB recommendations, pricing was set with a maximum bid per click of US $0.90 and maximum daily budget of US $25. Additionally, our LGBT-focused partner organization posted an announcement regarding the study on its website forum from November 3 to 7, 2012.

At a total cost of US $100, with an average of US $0.68 per click, the FB ad campaign resulted in 148 clicks and a CTR of 0.04 (Table 1). We spent an average cost of US $2.50 per enrolled participant (n=40).

In January 2013, another recruitment effort was implemented to enroll youth for the second set of focus groups. Facebook ads were submitted 5 days before the intended recruitment start date to ensure we had approval; ads were approved the same day of submission. The FB ads ran for 9 days (ie, January 14-22, 2013). Pricing was set again with a maximum bid per click of US $0.90 with a maximum daily budget of US $25. Another LGBT-focused partner organization also posted an announcement of the study on its FB page on January 12, 2013.

**Figure 1:**
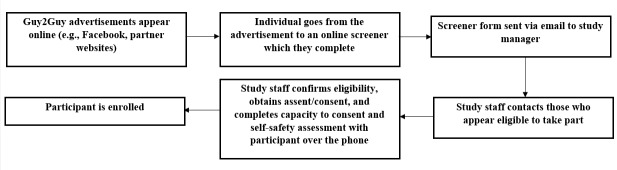
Guy2Guy flowchart.

A total of 13 screeners were received during the first 3 days of recruitment. Of these, 6 appeared to be eligible. To invigorate enrollment, our previous LGBT-focused organization partner emailed the recipients in its mailing list on January 16, 2013. We also modified the FB recruitment ads by removing the targeted interests or keywords (eg, Katy Perry), adding targeting criteria to include teenaged men who were “interested in men,” updating the image to one thought to be more relevant (Figure 1), increasing the daily ad budget to US $100, and changing the maximum bid per click to US $0.74. These changes doubled our selected audience (ie, the number of people the ad had the potential to reach based on ad targeting criteria) from 21,000 to 46,000 and resulted in 143 newly completed screeners in a 24-hour period.

The FB ads were active for a total of 7 days, posted nonconsecutively between January 14 and 22, 2013 (ie, ads were paused or stopped during the ad campaign depending on recruitment needs). We spent an average of US $6.96 per enrolled participant in this second effort (Table 1; n=40).

**Table 1:** Facebook advertisement placement metrics by study activity.

Facebook ad placement metrics by study activity	Placement	Impression device	Reach	Clicks	Unique clicks	CTR^a^	uCTR^b^	Cost^c^	CPC^c,d^	Cost^c^ per unique click	Cost^c^ per each enrolled participant
Focus group 1^e^ (n=80)											
	N/A^f^	N/A	93,823	148	148	0.04	N/A	$100.00	$0.68	N/A	$2.50
Focus group 2^g^ (n=80)											
	N/A	N/A	78,235	846	774	0.11	1.11	$278.35	$0.39	$0.42	$6.96
Content advisory team 1^h^ (n=24)											$32.52
	Right column ads on home page	Desktop or laptop	220,332	1667	1525	0.06	0.27	$483.99	$0.16	$0.18	
	Right column ads	Desktop or laptop	161,051	839	767	0.04	0.22	$296.54	$0.19	$0.21	
	Unknown placement	Other	335	0	0	0	0	$0.00	$0.00	$0.00	
Content advisory team 2^i^ (n=24)											$29.52
	Right column ads on home page	Desktop or laptop	52,184	418	381	0.07	0.51	$101.12	$0.21	$0.23	
	Right column ads	Desktop or laptop	37,327	168	157	0.05	0.35	$46.29	$0.22	$0.23	
	News Feed	Desktop or laptop	17,175	271	221	1.45	1.23	$95.44	$0.34	$0.42	
	News Feed	Mobile phone or tablet	133,202	1432	1256	1.82	1.51	$465.65	$0.33	$0.39	
	Unknown placement	Other	3	0	0	0	0	$0.00	$0.00	$0.00	
Beta test^j^ (n=20)											$17.19
	Right column ads on home page	Desktop or laptop	13,276	71	67	0.11	0.4	$24.36	$0.27	$0.28	
	Right column ads	Desktop or laptop	12,071	84	79	0.1	0.53	$35.05	$0.28	$0.28	
	News Feed	Desktop or laptop	7006	144	115	1.56	1.36	$40.90	$0.26	$0.30	
	News Feed	Mobile phone or tablet	34,886	1430	1031	3.13	2.35	$243.58	$0.23	$0.31	
RCT^k^ (n=302)											$12.54
	Right column ads on home page	Desktop or laptop	54,304	607	548	0.17	0.79	$455.27	$0.72	$0.77	
	Right column ads	Desktop or laptop	43,736	411	386	0.12	0.81	$346.01	$0.68	$0.72	
	News Feed	Desktop or laptop	34,234	728	607	1.36	1.28	$349.81	$0.57	$0.64	
	News Feed	Mobile phone or tablet	194,084	10,535	7333	3.54	3.12	$2635.36	$0.35	$0.42	

^a^ CTR: click-through rate.

^b^ uCTR: unique click-through rate.

^c^ All costs are in US dollars.

^d^ CPC: cost per click.

^e^ Facebook (FB) ad pricing structure: maximum bid per click of US $0.90. Daily budget: US $25.

^f^ N/A: not applicable (FB did not have the information available at the time of recruitment).

^g^ Initial FB ad pricing structure: maximum bid per click of US $0.90. Daily budget: US $25. Modifications to ad: 3 days into recruitment, the interest targeting was removed (eg, Katy Perry), criteria to target teenaged men “interested in men” was added, and the ad image to be more relevant to the population. Daily ad budget was also increased to US $100 and the maximum bid per click changed to US $0.74.

^h^ FB ad pricing structure: maximum bid per click of US $0.69. Daily budget: US $150. Modifications to ad: before launch of content advisory team 1 ads, multiple variations of the same ad, in which the headline and tagline were different, were created.

^i^ Daily budget: US $150. Modifications to ad: before launch of content advisory team 2 ads, pricing was modified to be optimized for clicks as opposed to preidentifying a maximum bid per click.

^j^ Daily budget: US $100. The same FB ad settings as described in the content advisory team 2 were used in the beta test effort.

^k^ RCT: randomized controlled trial. Daily budget ranged from US $50 to $100. Modifications to ad: during the RCT, FB added the option to select if one was attracted to “men or women,” which was integrated into the ad campaign (eg, allowing us to better target those that may identify as bisexual). Previously just the “men” or “women” options were available. New ad images were introduced toward the end of field that were thought to be more salient to specific populations (ie, younger participants, nonwhite race). Given the time span spent on recruitment ads were regularly adjusted to target based on age (eg, if we wanted to reach more 14-year-olds, ads were modified to specifically target those who are 14 years of age based on their FB profile).

#### Recruitment Efficiency

We received 209 completed screeners from the first round of focus groups, of which 94/209, 45.0% did not meet the study eligibility criteria. The most common reasons for ineligibility were age 41/94, 44%; not being cisgender male 20/94, 21%; and not being enrolled in an unlimited text messaging plan 12/94, 13%. For the second round of focus groups, we received 251 completed screeners, of which 99/251, 39.4% did not meet the study eligibility criteria. The most common reasons for ineligibility were not being a cisgender male 30/99, 30%; not being the exclusive owner of a cell phone 20/99, 20%; and not being enrolled in an unlimited text messaging plan 17/99, 17%.

Among the 115 candidates who appeared eligible from their Web-based screener responses during the first round of focus groups, 77/115, 67.0% candidates were contacted during the enrollment period. The remaining eligible candidates were not contacted because either the target sample size was met or their particular allocation bin was full; this applies for all study activities. It took 9 days to enroll 40 participants (ie, 20 sexually experienced, 20 sexually inexperienced) for the first round of focus groups. For the second round of focus groups, among the 152 candidates who appeared eligible given their screener responses, 75/152, 49.3% were contacted. Candidates were contacted until our target sample size of 20 participants was met for each focus group. The second round of focus groups required 11 days to enroll 40 participants.

### Content Advisory Teams

#### Advertisement Metrics

Next, we recruited a content advisory team, comprising 10 young participants from our target population, to review the draft of our program content. To do so, we made several changes to the FB campaign originally launched for the focus groups: we created multiple versions of the same ad in which the headline and tagline varied, increased the daily budget to US $150, and changed the maximum bid per click to be between US $0.25 and $0.69. The FB ads were live for 10 days between July 5 and 14, 2013. These changes resulted in an average cost of US $32.52 per enrolled participant (Table 1; n=24).

We also created a Google AdWords campaign of 187 keywords (eg, gay guys, gay teen, gay chat rooms). Ads were targeted by: network (all), device (all), location (United States), and language (English). The bid strategy was focused on clicks, manual maximum CPC bidding, and a set maximum daily budget of US $329 per day. A total of 3 different Google AdWords campaigns ran for 7 days (July 6-12, 2013) and received 962 clicks, 79,315 impressions, a CTR of 1.2%, and CPC of US $1.61. The total cost of the campaign, which was included in the advertising budget grant covered by Google AdWords, was US $1547.07.

After integrating findings from the first content advisory team into the content, we recruited a second content advisory team to confirm the modifications to the messaging. The FB recruitment campaign was further modified to optimize the pricing to get more clicks (ie, FB automatically bids to maximize the clicks to a website that can be achieved with the campaign budget) as opposed to preidentifying a maximum bid per click [26]. The FB ads were active for 7 days between August 28 and September 5, 2013, resulting in an average cost of US $29.52 per enrolled participant (n=24).

We used the same targeting criteria as described above in our Google AdWords campaign. As an exception, we set the network specifically to Google search networks (ie, a group of search-related websites, such as Google search sites and non-Google search sites—like AOL—that partner with Google to show search ads, where the ad may appear), rather than both search and display networks (ie, display networks consist of a group of more than a million websites, videos, and apps where the ad may appear) [27]. A total of 4 Google AdWords campaigns ran for 10 days (August 28 to September 6, 2013) using the same list of keywords noted above. Google AdWords received 2055 clicks, 209,257 impressions, a CTR of 0.98%, a CPC of US $1.59, and a total cost of US $3267.79.

#### Recruitment Efficiency

We received 271 Web-based screeners from the first content advisory team, of which 132/271, 48.7% did not meet the study eligibility criteria. The most common reasons for ineligibility were age 45/132, 34.1%, not being the exclusive owner of a cell phone 42/132, 31.8%, and not being enrolled in an unlimited text messaging plan 10/132, 7.6%. For the second content advisory team, 246 completed screeners were received, of which 84/246, 34.1% did not meet the study eligibility criteria. The most common reasons for ineligibility were not being enrolled in an unlimited text messaging plan 30/84, 36%; not being the exclusive owner of a cell phone 21/84, 25%; and having not used text messaging for 6 months or more 17/84, 20%.

Among the 139 eligible candidates based on the Web-based screener responses received during the first content advisory team, 79/139, 56.8% candidates were contacted during the enrollment period. For the second content advisory team, among the 162 eligible candidates, 69/162, 42.6% candidates were contacted. A total of 48 participants were enrolled in both content advisory teams. None of the participants were enrolled through the Google AdWords campaign; all were from FB outreach efforts.

It took 10 days to enroll 24 participants in the first content advisory team as well as the second content advisory team, including the time to schedule and complete the enrollment telephone calls.

### Beta Test

#### Advertisement Metrics

The same FB ad settings as described above in the second content advisory team were used in the beta test effort, with the exception of reducing the daily budget to US $100. The FB ads were active for 7 nonconsecutive days between March 9 and 29, 2014, and we spent an average cost of US $17.19 per each enrolled participant (Table 1; n=20).

In an attempt to increase the reach of our Google AdWords, 6 campaigns were created for the beta test recruitment effort. Additional headlines hypothesized to be more attention-grabbing (ie, by asking a question: “Are you a gay or bi teen?”) were added. Google AdWords were active for 6 days (March 12-17, 2014) and generated 599 clicks, 42,092 impressions, a CTR of 1.4%, CPC of US $1.43, and a total cost of US $854.46.

#### Recruitment Efficiency

We received 236 completed screeners during the beta test recruitment, of which 68/236 28.8% did not meet the study eligibility criteria at the initial screening. The most common reasons for ineligibility were not being the exclusive owner of a cell phone 21/68, 31%; did not intend to keep the current cell phone for 6 months or more 21/68, 31%; and not being cisgender male 15/68, 22%.

Among the 168 eligible candidates based on the Web-based screener responses received, 51/168, 30.4% candidates were contacted during the enrollment period; 40/51, 78.4% candidates responded; and 20/40, 50.0% participants were enrolled in the beta test. Again, no participants were enrolled through the Google AdWords campaign. It should be noted that the question querying how candidates heard about the research study was removed from the beta test Web-based screener. That said, no participants reported hearing about the study through Google AdWords.

It took 16 days to enroll 20 participants into the beta test. The length of time to complete enrollment exceeded that for previous recruitment activities because we newly implemented our targeting strategy to ensure a diverse sample of participants. This protocol was added at this step to ensure feasibility during the RCT enrollment effort.

### Randomized Controlled Trial

#### Advertisement Metrics

**Figure 2:**
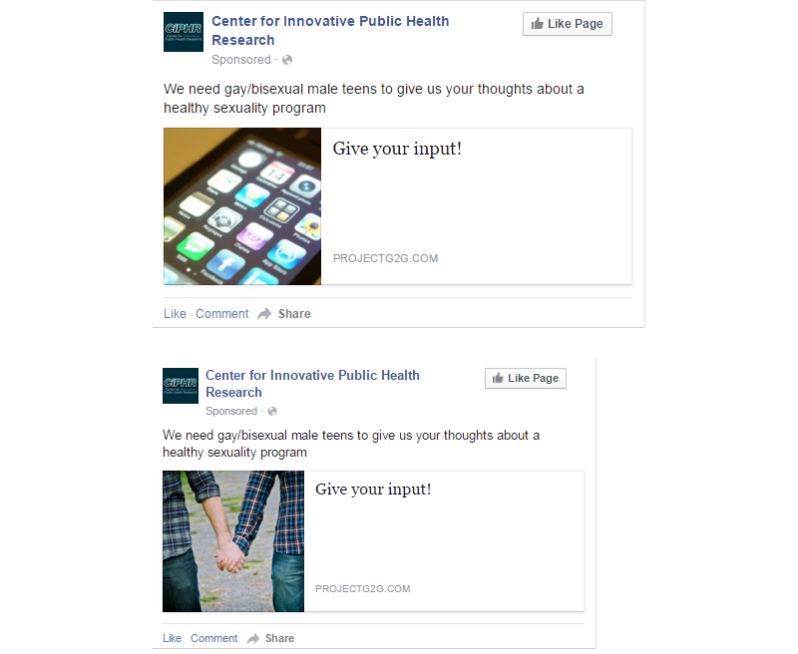
Comparison of the original versus modified focus group Facebook recruitment advertisements.

The same FB ad settings used in the beta test were implemented in the RCT. We were able to take advantage of a newly added FB targeting category that allowed us to advertise to users who were “interested in men and women”; previously, we were only able to target those who were “interested in men.” New ad images were used toward the end of field that were thought to be more salient to specific populations (ie, younger participants, nonwhite race; Figure 2, numbers 2-5). The FB ads were active for 52 nonconsecutive days between June 20 and October 31, 2014, and cost an average of US $12.54 to enroll each of the 302 RCT participants (Table 1). Notably, 32.3% (US $1221.55/$3787.08) of the recruitment money was spent to reach and enroll the last 10% of the sample, as these represented the youth who were particularly difficult to reach (eg, 14- to 15-year-old black youth; 14-year-old youth). Excluding these participants, the average cost per enrolled participant was US $9.47.

In total, 4 Google AdWords campaigns using the same targeting criteria as above were active for 15 days (June 16-30, 2014) before they were discontinued. The ads generated 1324 clicks, 132,843 impressions, a CTR of 1.00%, a CPC of US $1.59, and a total cost of US $2105.29.

#### Recruitment Efficiency

We received 1522 completed screeners during the RCT recruitment, of which 411/1522, 27.0% were ineligible based on their responses to the Web-based screener. The most common reasons for ineligibility were not being cisgender male 129/411, 31.4%; did not intend to keep current cell phone for 6 months or more 108/411, 26.3%; and not being enrolled in an unlimited text messaging plan 77/411; 18.7%. Among the 1111 eligible or potentially eligible screeners received, 600/1111, 54.0% candidates were contacted sequentially based on their allocation bin during the enrollment period. Those who were eligible but their allocation bin was filled were not contacted. Among those contacted, 494/600, 82.3% candidates responded, and 342/494, 69.2% candidates spoke to study staff, ultimately resulting in the enrollment of 328/342, 95.9% participants, of whom 302/328, 92.1% were randomized into the RCT.

It took 148 days to enroll and randomize 302 participants at an average rate of 13.7 participants per week (range 1-28 participants). Research staff aimed to enroll between 15 and 20 participants per week, so that a manageable number of participants were actively receiving the intervention at any given time. An average of 1.54 contact attempts were required to enroll study participants (range 1-6).

#### Sample Diversity

Screeners were more commonly completed by sexually experienced 865/1522, 56.8% than inexperienced 628/1522, 41.3% young men. Inexperienced youth were also slightly more likely to respond to staff outreach about the program, 258/494, 52.2% versus 236/494, 47.8%. In total, 1036/1522, 68.1% of the screeners were from white youth, compared with 91/1522, 5.98% from black or African American youth and 39/1522, 2.6% from Asian youth.

**Figure 3:**
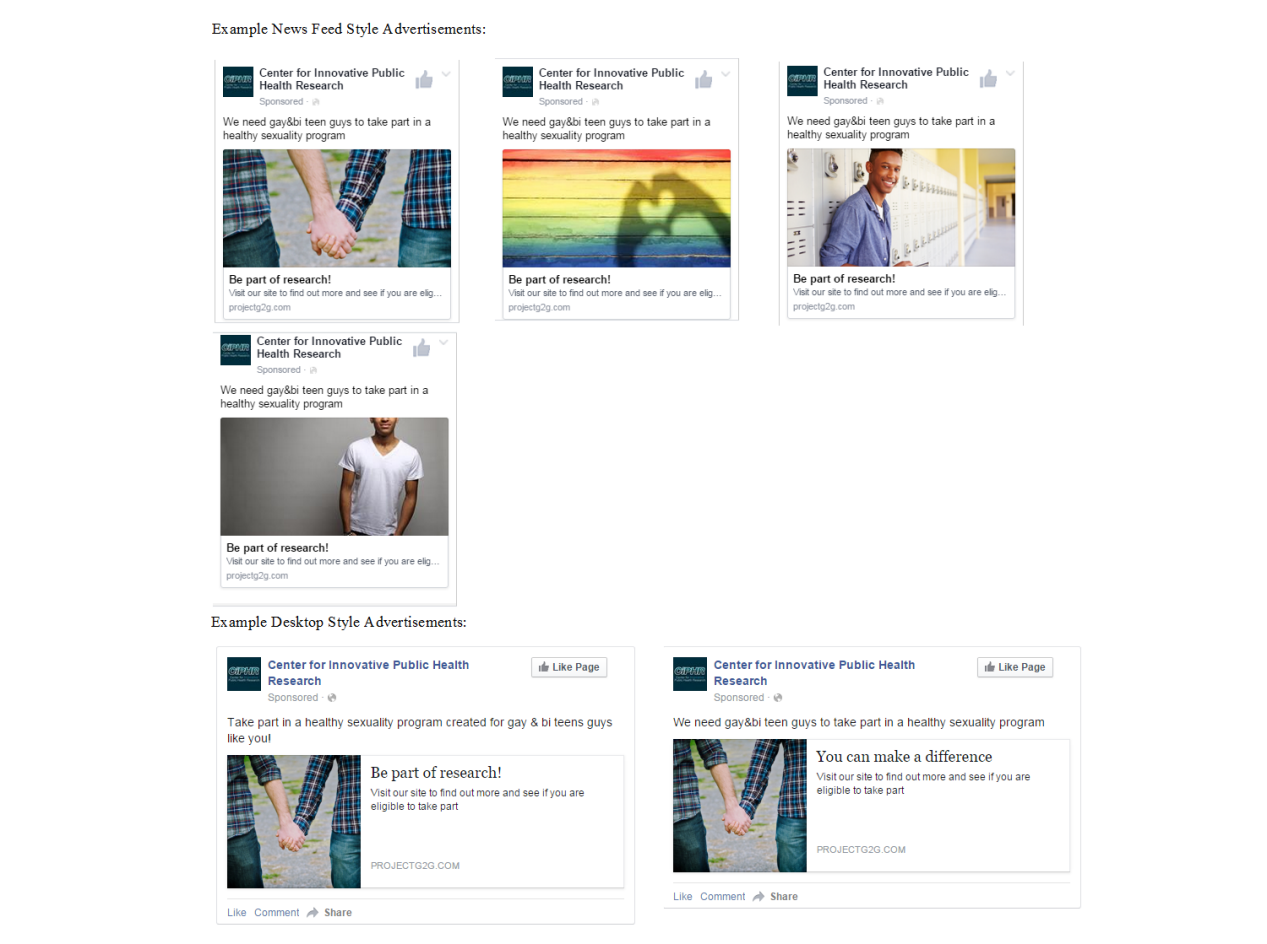
Example of Facebook recruitment advertisements used during the randomized controlled trial.

Facebook advertising was regularly adjusted based on the allocation bins (Figure 3). For example, if all the bins for 18-year-olds were filled, we adjusted the targeting on FB ads to include only 14- to 17-year-olds. Also, when noticeably fewer screeners were received from 14- to 15-year-olds, in part because of fewer FB profiles of young people in this age range, we created FB ads that specifically targeted these younger teenagers. This purposeful sampling strategy (Table 2) resulted in a sample that was 14.2% black or African American and 23.2% living in rural settings.

**Table 2:** Randomized controlled trial randomized participant demographic characteristics based on allocation bins (N=302).

Participant characteristics^a^		n (%)
**Sexually experienced**		
	Yes	153 (50.7)
	No	149 (49.3)
**Age, years**		
	14-15	116 (38.4)
	16-18	186 (61.6)
**Race**		
	White	204 (67.5)
	Black	43 (14.2)
	All other	55 (18.2)
**Hispanic**		
	Yes	67 (22.2)
	No	235 (77.8)
**Sexual identity**		
	Gay and/or queer	195 (64.6)
	Bisexual	107 (35.4)
**Type of community**		
	Rural	70 (23.2)
	Urban	232 (76.8)

^a^Demographic characteristics reflect participant responses during the phone screener, as most of these questions were not again queried in the baseline survey. With exception, sexual experience was assigned based on response to the baseline survey as a more comprehensive battery of questions were asked that would subsequently lead to participants being assigned to the sexually experienced or sexually inexperienced program.

**Figure 4:**
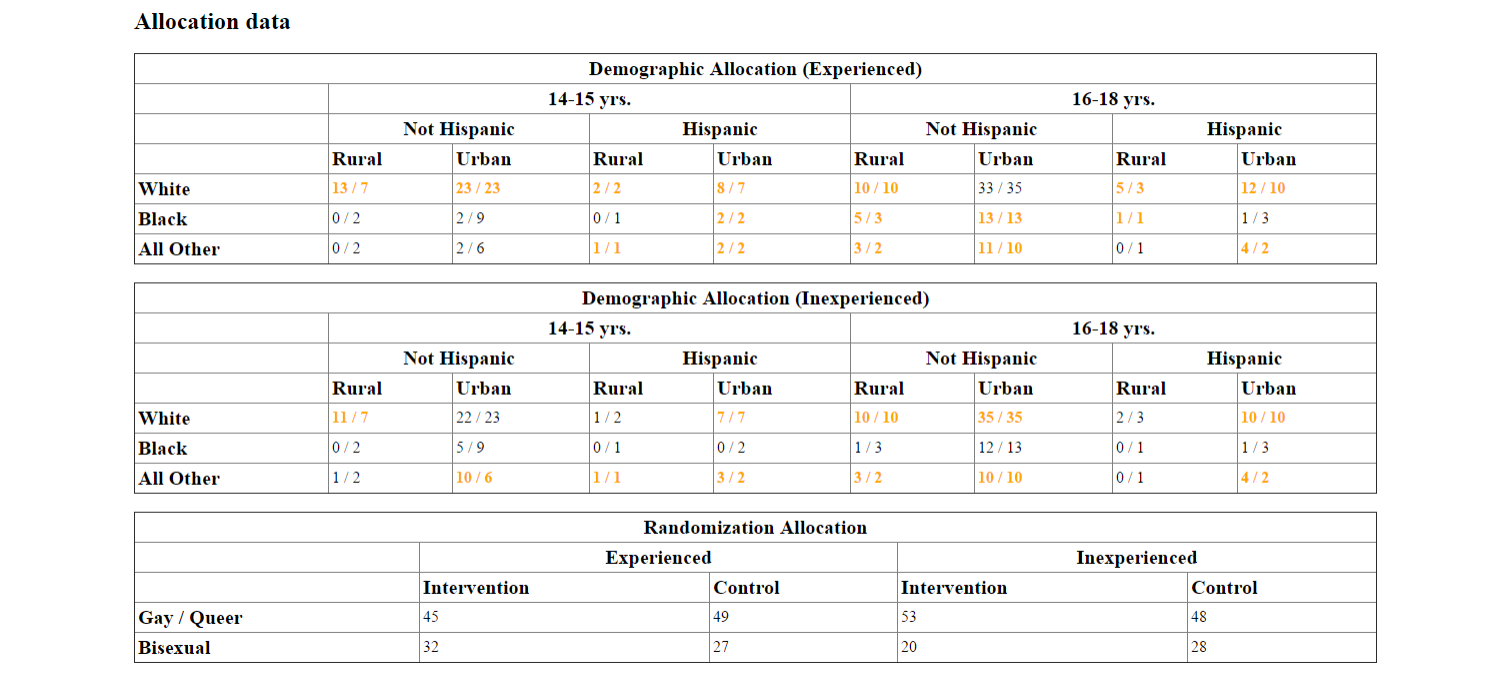
Example of real-time enrollment monitoring tool on the Guy2Guy study administrative interface.

Challenges in balancing the sample were encountered when participants provided different responses on the phone or baseline survey than what they had reported in the Web-based screener, resulting in the allocation bin being overfilled (Figure 4). For example, although the same behaviorally based items to query sexual experience were used in the Web-based screener and baseline survey, 29 participants indicated a different sexual experience history than they had first reported in the Web-based screener. Similar issues were also observed with other demographic characteristics used for allocation (eg, race, n=38; ethnicity, n=22).

One Facebook advertisement image used is excluded because of copyright permissions. The image depicted 2 African American teens—one with his arm around the other.

Within each bin, the number on the right is the target sample size, the number on the left is the number currently enrolled. Black font reflects a bin that has not yet reached its preset target sample size. Orange font reflects a bin that has reached or gone over the target sample size. For example, 13/7 signifies that 13 white, rural, non-Hispanic, sexually experienced 14- to 15-year-olds were enrolled, but the target based on the allocation bin was 7.

## Discussion

On the basis of our iterative development of an Internet-based recruitment strategy aimed at 14- to 18-year-old AGBM, our findings suggest that protocols designed to use FB to reach a national sample of diverse youth will be most effective if they use images salient to the target population, are broad (eg, targeted based on minimum eligibility criteria, such as age, sex) rather than too specific (eg, not targeted on interests or keywords), are regularly monitored for performance, and the ad targets are regularly modified to meet recruitment needs.

Our findings suggest that not all Web-based advertising has the same effect when trying to reach AGBM adolescent men. Indeed, our Google AdWords outreach effort did not result in a single participant being enrolled. This is likely because our reach—based on the keyword targeting we utilized—was too diffuse. Beyond keywords, we could only target Google AdWords campaigns by location (eg, United States). Therefore, the people who saw our ads went well beyond our very specific population of interest. On the other hand, FB advertising was much more successful, resulting in the enrollment of 405/450, 90.0% of our study sample across the 4 research activities. The FB ad manager allowed the ad to be targeted on multiple criteria beyond location, such as gender, age, and the relationship interests that FB users indicate in their profile (ie, interested in men, interested in men and women). In sexual health research, this ability to target ads based on relationship interest can narrow the ad audience down to the target audience, in our case AGBM, thereby increasing recruitment cost-effectiveness and efficiency. It is worth noting that our experiences may not generalize to other populations and research topics, however [28,29].

Although our specific recruitment goals were identified before recruitment (eg, race, ethnicity, age), the ability to reach recruitment goals is nonetheless dependent on having those from diverse backgrounds actually see the recruitment advertisement and subsequently complete a screener. Although FB ad manager did not allow for tailoring based on diversity targets (eg, race, ethnicity) at the time, resulting screeners reflected a sufficiently diverse group of youth that allowed us to follow-up with youth who met our diversity goals. It should be noted that updating the ad image so that it better resonates with a specific population or targeting the ad to specific cities or states that have demographic profiles of interest can also invigorate sample diversity.

### Limitations

These findings should be considered within the context of the study’s limitations. For example, by targeting FB users through the “interested in” targeting category, we targeted those youth who are more likely out because they indicated their dating preferences on a social networking profile. However, FB users can choose to make specific parts of their profile private (eg, only visible to them or their friends), so that they can still complete their profile while limiting who is able to see their information (eg, parents, nonfriends). Additionally, by recruiting on FB, this inherently excludes those youth who are not on FB. Given that FB continues to be the most popular social networking site for teenagers, however [30], this recruitment method still provides a unique opportunity to reach a national sample of adolescents. Indeed, some may wonder why we did not use other Web-based recruitment strategies such as the Turk. This is because our goal was to test the interest of our intervention among AGBM teenagers in places where they “hang out” online rather than places where people go to earn money.

### Conclusions

This research has elucidated important and effective strategies for utilizing social media for the recruitment of youth to research. The utilization of social media for recruitment of hard-to-reach youth, particularly sexual minority teenaged males for whom privacy may be an issue, is increasingly important as targeting these youth for health, support, and sexual health programs is a public health imperative. Although our research indicates the cost-effectiveness and overall efficiency of using Facebook in particular for recruiting AGBM into research, alternative social media strategies should be explored as they become more popular with youth. It will be essential to continue documenting successful outlets for recruitment of hard-to-reach youth. Given that the research project we described herein was sensitive in nature and we were able to demonstrate success in Web-based recruitment, we are confident that these strategies can be utilized to recruit AGBM for research related to sexual health topics and perhaps other topics. Additional research is needed to demonstrate the success of these strategies with other populations.

## Abbreviations

AGBM: adolescent gay, bisexual, and other teenaged men who have sex with men
CPC: cost per click
CTR: click-through rate
FB: Facebook
HIV: human immunodeficiency virus
IRB: institutional review board
LGBT: lesbian, gay, bisexual, and transgender
RCT: randomized controlled trial
